# Determinants of access to health facilities among under-five children with caregiver-reported fever in the context of seasonal malaria chemoprevention in Togo, 2020–2022

**DOI:** 10.1186/s41182-025-00717-5

**Published:** 2025-03-11

**Authors:** Endeshaw Degie Abebe, Sikai Huang, Kevin Baker, Fantche Awokou, Meseret Zelalem, Tadesse Shiferaw Chekol, Abebe Tilaye Weldemichael, Sol Richardson

**Affiliations:** 1 Vanke School of Public Health, Tsinghua University, Beijing, 100083 China; 2 Ministry of Health, 2P8W+GP6, Sudan St, Addis Ababa, Ethiopia; 3 Malaria Consortium UK, the Green House, 244–254 Cambridge Heath Road, London, E2 9DA UK; 4 Department of Global Public Health, Karolinska Institute, Stockholm, Sweden; 5 Malaria Consortium Togo, Malaria Consortium, Quartier Administratif, Rue Adamé, Lomé, Togo; 6 Addis ECD Centre for Excellence, Learning and Innovation, Addis Ababa, Ethiopia; 7 Armauer Hansen Research Institute, PO Box 1005, Addis Ababa, Ethiopia

**Keywords:** Malaria, Access to health services, Seasonal malaria chemoprevention, Togo

## Abstract

**Background:**

Malaria is responsible for 580,000 deaths among children under 5, or 95% of all malaria deaths per year globally. Seasonal Malaria Chemoprevention (SMC) is a malaria control intervention in Togo and other African countries targeting children under 5 years old during the peak malaria transmission season. Delaying access to healthcare for children with malaria can result in serious health problems, including heightened morbidity and mortality, complications related to cerebral malaria and anemia, as well as impaired cognitive development. This study aimed to identify determinants of access to health facilities for children with caregiver-reported fever, in the context of SMC campaigns in Togo.

**Methodology:**

We analyzed data from three representative annual end-of-round SMC surveys on SMC-eligible children aged 3–59 months residing in the provinces of Savanes, Central and Kara in Togo, conducted during 2020–2022. We performed a descriptive analysis and fitted logistic regression models to assess predictors of health facility access. Our sample included all children with a caregiver-reported fever in the month before the survey. Model variables included household distance to their local health facility, quintiles of household wealth, household visit by SMC distributors in the previous month, household nomad status, literacy of primary caregivers, and the age and sex of both eligible children and their primary caregivers.

**Results:**

Our analytic sample included 6,252 SMC-eligible children, including 1,418 experiencing fevers. Most children with fever (62.6%, 95% CI 60.0–65.0%) accessed health facilities. Adjusted odds ratios and 95% confidence intervals obtained from the logistic regression analysis found a statistically significant linear relationship between children's adjusted odds of access to health facilities and their distance from the nearest facility, with 2% lower odds of access for each additional kilometer of distance (AOR = 0.98, 95% CI 0.97–0.99). Households with SMC distributor visits were significantly more likely to access health facilities (AOR = 2.20, 95% CI 1.22–3.96). Children of female primary caregivers had higher odds of access (AOR = 1.42, 95% CI 1.05–1.93).

**Conclusion:**

Febrile children’s access to malaria testing and treatment in Northern Togo requires further improvement, particularly among those further from health facilities and with lower household wealth.

## Background

Malaria is responsible for 580,000 deaths among children under five, representing 95% of all malaria deaths worldwide in 2022 [1]; *Plasmodium falciparum* (PF) is the primary cause of malaria in high malaria-endemic regions like the Sahel region of sub-Saharan Africa (SSA) [2]. Seasonal Malaria Chemoprevention (SMC) in Togo targets children aged 3–59 months during peak malaria transmission. Coverage across most countries with SMC delivery has significantly improved from 2013 to 2020, reaching 80% in Togo and higher rates in nearby countries: 93–97% in Chad, 90% in Nigeria, 94% in Burkina Faso, and 93% in Mozambique. These increases in coverage have contributed to a notable reduction in overall malaria incidence and complications compared to previous years, with confirmed cases in Togo dropping from 11,269 in 2016 to 1,395 in 2020 [3].

Achieving global malaria control targets and improving child health outcomes require an understanding of the factors that influence children’s access to medical facilities for malaria treatment [4]. Delaying access to health services for children with malaria can lead to severe health issues such as increased morbidity and mortality including cerebral and anemia-related complications, and impaired cognitive development [5]. Ensuring protection against malaria infection also depends on quality of services delivered [6].

Access to healthcare facilities is a human right for children who are vulnerable to malaria, which poses serious public health threats, especially in SSA [7]. Rural children often face challenges in receiving treatment at health facilities due to limited access to high-quality healthcare facilities and services [8]. Socioeconomic factors, such as poverty, healthcare disparities, and illiteracy, can potentially impede access to healthcare. Addressing social and economic health disparities in access to malaria interventions and treatment for children under 5 years of age is crucial to reducing malaria-related morbidity and mortality in Togo and elsewhere [9].

Children in other SSA countries living near healthcare facilities are more likely to receive malaria treatment and caregivers from wealthier families are more likely to seek healthcare for illnesses for children compared to those from less wealthy households, which could also be influenced by their maternal education level [10]. Despite decreases in incidence of severe malaria in various SSA countries, access to healthcare facilities for the treatment of malaria, however, is still an ongoing issue [11]. Togo's health system, which operates on a decentralized model, focuses on primary healthcare services mainly provided by public facilities. The Ministry of Health implements policies to achieve universal health coverage (UHC) and enhance access for vulnerable populations. Key initiatives include health insurance schemes and community health programs targeting maternal and child health, infectious diseases, and nutrition. However, the distribution of health facilities is uneven, with urban areas generally having better access than rural regions. Geographic barriers and inadequate infrastructure complicate access, prompting the government to ensure that health facilities are located within a specific radius of communities, emphasizing local engagement in planning [12].

SMC, meanwhile, involves delivering antimalarial medicines to populations who are at risk during the peak of the malaria season. SMC, typically consisting of amodiaquine and sulfoxide-pyrimethamine to prevent PF malaria in children aged 3–59 months, has been supported by Malaria Consortium in several countries with highly seasonal malaria transmission [7, 13]. In 2013, Togo introduced SMC to reduce malaria burden during peak transmission months. It provides monthly treatment courses for children aged 3–59 months at the start of the high-transmission season using sulfadoxine-pyrimethamine (SP) and amodiaquine (AQ), aiming to maintain sufficient drug concentrations to provide protection between monthly delivery cycles, as one dose of SP + AQ plus and two daily doses of AQ. SMC is delivered door-to-door by trained community distributors, with the first dose given as a direct observed therapy. The remaining AQ doses are left for daily administration by children’s caregivers. In Togo, during 2020–2022, SMC was delivered in annual rounds each with four monthly delivery cycles covering July to October [14]. A longitudinal study conducted from 2013 to 2020 in Togo reported a decrease in confirmed malaria cases from 11,269 in 2016 to 1,395 in 2020, demonstrating the effectiveness of SMC in reducing malaria incidence among children under five. The study also indicated that the effectiveness of SMC ranged from 76.6% to 96.2% across different treatment rounds [15].

### Aims and objectives

To our knowledge, no previous study has attempted to investigate determinants of access to health services among febrile children specifically in SMC settings or in Togo, or to assess the association between variables related to SMC (such as household visits by SMC community distributors) and health facility access.

This study aimed to identify determinants of access to a health facility among children with caregiver-reported fever in the context of SMC delivery in Togo during 2020–2022. Specific objectives were to describe the analytic sample of SMC-eligible children in Togo in caregiver-reported fever events in the last month and reasons for not accessing health facilities, and to conduct a regression analysis to identify child, caregiver, household, and area-level factors associated with health facility access, including distance to health facilities.

## Methods

### Study population

Data were obtained from end-of-round SMC coverage surveys following the final annual cycle of SMC delivery (cycle 4 in Togo, delivered in October of each year). Surveys were conducted by independent investigators commissioned by Malaria Consortium in Togo from December 10–20 in 2020, October 22–30 in 2021, and October 10–20 2022, in three northern regions of Togo (Centrale, Kara, and Savanes) encompassing 19 districts where SMC was delivered. SMC was first delivered in Togo in 2013, and has been supported by Malaria Consortium since 2020. The study area, located at the boundary between tropical savannah and semi-arid Sahelian climatic zones, has high seasonality of malaria transmission. In 2021, it was estimated that an average of 489,389 children in the three regions aged 3–59 months, comprising an estimated 19.9% of the total population, was eligible for SMC per monthly delivery cycle [14].

Surveys had a target sample size of 2000, with 10 households sampled in 200 communities. To create a self-weighting sample, communities were chosen at random from a national sampling frame with a probability proportional to the population size in each community. Households were then sampled using a simple random method from local household lists and were included in the survey if they had one or more eligible children aged 3–59 months. In each household, a roster of all eligible children was made; one child was selected at random by the data collection application, and all survey questions related to that child, their primary caregiver, and their household. Further information on survey methods is available elsewhere [14].

### Data collection

Data were collected offline using Survey CTO version 2.70 on mobile devices by pairs of data collectors and uploaded daily to a remote server. Data collectors underwent standardized training, including field testing, to ensure uniform administration of the survey. Additionally, built-in logic checks within the SurveyCTO platform flagged inconsistent responses during data entry, prompting clarification where needed. Survey responses were recorded using pre-defined categories. Data on distances were obtained from the national survey sampling frame based on Ministry of Health estimates of travel distances between communities and their assigned local health facility, and merged with household-level data. Data were monitored daily for potential quality issues by a local data manager. In addition, location of data input was validated using GPS to ensure that the correct households were visited; surveys also included hidden variables allowing the data manager to ensure that sufficient time was taken for individual questions during household interviews.

### Inclusion criteria

The sample comprised children aged 3–59 months at the start of the annual SMC round and residing in the study provinces in Togo at any time during the SMC round; HIV-positive children, those with a known allergies to sulfa drugs, and children whose caregivers did not provide consent to participate in the survey, were excluded from the sample.

### Study variables

The dependent variable in our statistical analysis was children’s access to health facilities (“did [your child] attend a health facility after experiencing fever”); this question was only asked if children experienced a fever in the last 30 days preceding distribution of SP and AQ in cycle 4 (in October of each year) as reported by their primary caregiver (binary outcome: yes/no). Caregivers whose children did not attend health facilities were invited to provide reasons for non-attendance (response categories included “clinic too expensive”, “clinic too far”, “fever considered not serious”, “caregiver preferred alternative treatment”, and any other reason). Variables were selected from those available in the existing end-of-round dataset, with the widest-possible range of relevant variables considered for inclusion in our final model [16]. We considered the following independent variables as potential predictors of health facility access, grouped into child, caregiver and household characteristics: child sex (categorical: male/female), child age at the time of the survey (categorical: < 1 year/1 year/2 years/3 years/4 years/5 years), caregiver self-reported literacy (categorical: literate/illiterate), caregiver age (categorical: < 20 years/20–29 years/30–39 years/40–49 years/50–59 years/≥ 60 years), caregiver sex (categorical: male/female), self-reported caregiver level of education (categorical: none or only pre-primary education/informal or religious education/primary education/secondary education/higher education), head of household reported literacy (categorical: yes/no), household nomad status (categorical: yes/no), and household visited by an SMC community distributor for distribution of SMC medicines in cycle 4 (categorical: yes/no).

To analyze the distribution of the population wealth quintiles, we calculated household wealth scores by summing across the ten variables included in the Simple Poverty Scorecard^®^ for Togo and divided our sample of households into quintile categories (poorest, poor, middle, richer, and richest) [17]. Distance from households was defined based on travel distances between community clusters and their assigned health facility, using merged data obtained from the national survey sampling frame and based on health facilities assigned to each community, and operationalized as a continuous variable in units of kilometers (km).

### Caregiver-reported fever among SMC-eligible children

In this study, the confirmation of fever among children was based on caregiver reports rather than laboratory diagnostics such as rapid diagnostic tests (RDT) or microscopy; in Togo, confirmation of clinical malaria in primary care relies exclusively on the former. Caregivers were asked whether the child had experienced a fever within a specified timeframe, and their responses were used to assign presence of fever for the purposes of analysis.

### Descriptive analysis

Child, caregiver, and household variables were summarized using counts and percentages. A histogram was generated to show the distribution of distances from community clusters by travel distance to their assigned health facility.

### Statistical analysis

We fitted random-effects multivariate logistic regression models with random intercepts for the district, to identify predictors of children’s access to health facilities. Variables were selected for inclusion in a final model using a forward stepwise regression approach based on Collett’s method [16, 18]. Each variable was added to the model in turn and then regressed on the dependent variable. Variables considered for inclusion in the final model were retained in the model if the likelihood ratio test showed that their inclusion improved the model's goodness-of-fit (p < 0.05). Results were expressed as adjusted odds ratios (AOR), with 95% confidence intervals (95% CI) and p-values. Statistical significance was defined as p < 0.05, corresponding to a 95% confidence level.

We conducted regression diagnostics including the Hosmer-–Lemeshow test to evaluate the goodness-of-fit, and calculation of variance inflation factors (after refitting the final model as a fixed-effects model) to test for collinearity. We also considered whether the association between distance between households and health facilities, and health facility access, was linear. We added a quadratic term for distance to the final model; this was not statistically significant, however, suggesting that the non-linear component did not improve model fit.

## Results

### Descriptive analysis

Table 1 presents a descriptive analysis of variables related to children, caregivers, and households considered for inclusion in our statistical analysis. Of our sample of 6,252 SMC-eligible children, of whom 2,033 (32.52%) were sampled in 2020, 2,016 (32.25%) in 2021 and 2,203 (35.24%) in 2023, 1,418 of these (22.68%) experienced fever within the 30 days before the survey as reported by caregivers. Of those children experiencing fever, these 887 of 1,418 (62.55%) obtained access to health facilities. Among the remaining 531 febrile children who did not access health facilities, 211 (39.70%) caregivers reported being too far from a clinic, 170 (32.00%) reported choosing an alternate treatment for their child, 76 (14.20%) reported a belief that the fever was not a serious condition, 54 (10.00%) reported a belief that clinic attendance was too expensive, and 20 (4.00%) gave any other reason for lack of access to a health facility.

**Table 1:** Summary sample statistics (*N* = 6,252), end-of-round SMC coverage surveys, 2020−2022

Variable type	Variable category		Frequency (n)	Percentage (%)
Child outcomes	Child fever	Yes	1418	22.68
		No	4834	77.32
	Child’s access to a health facility if having fever	Yes	887	62.55
		No	531	37.45
	Why child did not access health facility	Clinic too expensive	54	10.00
		Clinic too far	211	39.70
		Fever considered not serious	76	14.20
		Caregiver preferred alternative treatment	170	32.00
		Any other reason	20	4.00
Child characteristics	Child’s sex	Male	3251	52.00
		Female	3001	48.00
	Child’s age	< 1 year	645	10.32
		1 year	1018	16.28
		2 years	1255	20.07
		3 years	1383	22.12
		4 years	1495	23.91
		5 years	456	7.29
Primary caregiver characteristics	Caregiver’s self-reported literacy	Literate	3305	52.86
		Illiterate	2947	47.14
	Caregiver’s age	< 20 years	468	7.48
		20–29 years	2137	34.20
		30–39 years	2514	40.21
		40–49 years	655	10.48
		50–59 years	254	4.06
		≥ 60 years	224	3.58
	Caregiver sex’s	Male	935	14.96
		Female	5317	85.04
	Caregiver’s level of education	No or pre-primary education	2971	47.52
		Informal or religious education	63	1.01
		Primary school	1471	23.53
		Secondary school	1572	25.14
		Higher education	175	2.80
Household characteristics	Wealth quintile	1 (poorest)	1451	23.21
		2 (poor)	1268	20.28
		3 (middle)	1402	22.42
		4 (richer)	1034	16.54
		5 (richest)	1099	17.58
	Head of household reported literacy	Literate	2879	61.83
		Illiterate	1777	38.17
	Nomad status of household	Yes	579	9.26
		No	5671	90.74
	Compound visited by community distributor (cycle 4)	Yes	5854	93.36
		No	398	6.64

**Fig. 1:**
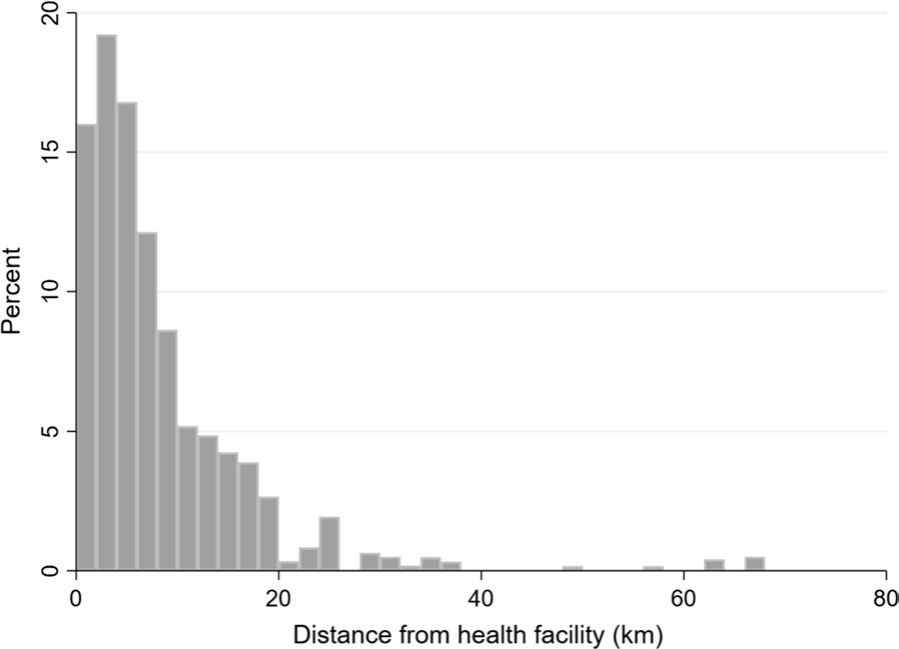
Distribution of respondents’ households relative to health facilities by distance in km. Figure displays a histogram showing the percentage distribution of households by distance to the nearest health facility, grouped in 2 km increments. Figure shows the distribution of health facilities relative to respondents’ households by distance in km, with bars showing increments of 2 km. Median distance from households to health facilities was 5 km (interquartile range: 3–10 km)

Regarding the age distribution of our total sample of 6,252 eligible children, 645 (10.32%) were aged 3−11 months (< 1 year), 5,151 (82.39) were aged 1−4 years, and 456 (7.29%) were aged 5 years at the time of the survey (eligible for SMC in cycle 4 as they were aged 3–59 months at the start of the annual SMC round). Figure 1.

### Statistical analysis

The results of the multivariate regression analysis, shown in Table 2, indicated that, after mutual covariate adjustment, children with a female primary caregiver had a 42% higher odds of health facility access compared to those with a male primary caregiver (AOR = 1.42, 95% CI 1.05–1.93). As the age of a primary caregiver increased to 50–59 years, the odds of children’s access to health facility were 60% lower (AOR = 0.40, 95% CI 0.17–0.93) compared to those with caregivers under 20 years. Children who had their home compound visited by community distributors for distribution of SMC medicines had significantly higher odds of access to health facilities (AOR = 2.20, 95% CI 1.22–3.96) than children whose compound had not. There was a statistically significant linear relationship between children's access to health facilities and their distance from their assigned health facility after covariate adjustment; for each additional km of travel distance from the household to the health facility, there was a 2% reduction in odds of health facility access compared with children living < 1 km from the health facility (AOR = 0.98, 95% CI 0.97–0.99).

**Table 2:** Results of a multivariate random-effects regression model for predictors of febrile children’s clinic access, end-of-round SMC coverage surveys, 2020–2022

Variable type	Variable category	AOR	95% CI	p-value
Child characteristics	Child’s age			
	<1year	0.79	0.52–1.20	0.267
	1 year	1.26	0.88–1.80	0.199
	2 years	1.10	0.79–1.55	0.544
	3 years	Reference		
	4 years	1.85	0.62–1.18	0.350
	5 years	1.19	0.75–1.88	0.462
	Child’s sex			
	Male	0.92	0.75–1.15	0.459
	Female	Reference		
Primary caregiver characteristics	Caregiver’s age			
	<20 years	Reference		
	20–29 years	0.62	0.33–1.14	0.125
	30–39 years	0.67	0.36–1.26	0.215
	40–49 years	0.70	0.35–1.42	0.323
	50–59 years	0.40	0.17–0.93	0.033
	≥60 years	0.34	0.10–1.22	0.100
	Caregiver’s sex			
	Male	Reference		
	Female	1.42	1.05–1.93	0.024
Household characteristics	Wealth quintile			
	1 (poorest)	Reference		
	2 (poor)	1.17	0.84–1.62	0.349
	3 (middle)	1.12	0.82–1.53	0.484
	4 (richer)	1.25	0.88–1.77	0.205
	5 (richest)	1.36	0.94–1.96	0.098
	Nomad status			
	Yes	0.96	0.67–1.39	0.847
	No	Reference		
	Compound visited by community distributor (cycle 4)			
	Yes	2.20	1.22–3.96	0.008
	No	Reference		
	Distance (km)	0.98	0.97–0.99	0.011

The Hosmer–Lemeshow test indicated appropriate model goodness-of-fit. Variance inflation factors for all variable categories included in the final model were all below 2, indicating very low multicollinearity between included variables.

## Discussion

Children’s health access in SSA countries is influenced by various factors, including geographical location, socioeconomic factors, healthcare infrastructure, and cultural beliefs [19]. SSA countries have made many efforts to improve child health access, including strengthening health infrastructure, increasing healthcare funding, improving healthcare workforce capacity, and promoting community-based healthcare services [20]. In addition to these efforts, SMC has been delivered in several countries, including Togo, to reduce the burden of malaria among young children during this seasonal high-transmission period. It is arguable that the intervention contributes to improving access by providing information about malaria treatment and health facility access for targeted children, given the association between household visits by distributors and health facility access [21].

A number of specific findings can be drawn from this study. First, our study described the proportion of SMC-eligible children accessing health facilities in Togo if experiencing fever; this was higher than that in another study undertaken in Togo in 2014 among children under five years of age covering all regions of the country (62.6% vs. 38.9%) [22]. It is not possible within the scope of this study to determine, however, whether the differences in the outcome between the studies can be attributable to impacts of policies implemented between 2014 and 2022. Second, we found that coverage of SMC household visits by distributors in the study area was high, at 93.36%, suggesting successful program implementation. Third, our study identified caregiver-reported reasons for children’s lack of access to health facilities, with the most frequent including “clinic too far”, “fever considered not serious”, and “caregiver preferred alternative treatment”. Fourth, we identified a range of child, caregiver, and household variables associated with health facility access; notably, these included caregiver’s age, household visit by SMC distributors, and distance from the household’s assigned health facility Children of older caregivers aged 50–59 were significantly less likely to access health facilities compared to those with caregivers under 20 years age; older caregivers may be less likely to take their children to a health facility when they are ill, possibly due to factors including reduced mobility, preference for traditional remedies, reduced decision-making power, and perceived barriers [23]. The odds of children’s access to a health facility while having fever were higher by 42% among those whose caregivers were female as compared with male. These findings suggest that the gender of the caregiver plays an important role in the health-seeking behavior for children with fever [24].

Children whose home compounds were visited by community distributors had a greater likelihood of accessing health facilities compared to those who did not receive such visits. This suggests that household interactions with seasonal malaria chemoprevention (SMC) distributors play a significant role in promoting access to malaria treatment. The increased access may be attributed to the information and support provided by the SMC community distributors, as well as their referrals of febrile children to appropriate health facilities.

Each additional km of distance from the household to the assigned health facility was associated with a 2% lower odds of health facility access compared with children living < 1 km from the health facility. This result is consistent with a study in Burkina Faso on care-seeking for febrile under-five children, which reported a 13% reduction in odds of health facility access among those living ≥ 2 km from health facilities compared with those living within 2 km [25]. This may be attributed to families without access to transportation finding long walks to healthcare facilities physically demanding, particularly when caregivers are also carrying sick children.

In summary, the findings from this study not only highlight existing disparities and barriers in healthcare-seeking behavior but also provide actionable insights that can guide future policies and interventions to enhance access to health facilities for children with fever in Togo. At the same time, they highlight the positive role of SMC community distributors in promoting healthcare access. By addressing these determinants, stakeholders can improve health outcomes and reduce the burden of malaria among vulnerable populations.

### Strengths and limitations

One strength of this study was its use of a large, readily available end-of-round SMC coverage survey database with a wide range of relevant variables based on high-quality surveys conducted by third-party contractors not involved in SMC implementation. Surveys employed in this study were designed to be representative of all eligible children, and their caregivers and households, in the three regions included in the study. The study had limitations, however, such as reliance on self-reporting for collection of data on most study variables; responses for some variables such as caregiver literacy may have been subject to biases such as social desirability bias. In 2020, the first year SMC was supported by Malaria Consortium, there was a time delay of around 10 weeks between delivery of SP and AQ in cycle 4 and data collection; this may have reduced reliability of reporting due to potential recall bias. This issue was remedied in 2021 and 2022, in which all data were collected in the same month at the final SMC cycle. Children’s fever, as reported by the caregiver, may not have been the result of malaria, and alternative causes could have been involved. Furthermore, no data were available on the severity of fever. Another limitation was the range of variables available for analysis, which, unlike a previous study in Togo, did not include qualitative perception of difficulty of reaching the nearest health facility, childcare behaviors such as breastfeeding, and religious affiliation [21]; residual confounding may therefore have occurred as not all potential predictors of health facility access could be included in the surveys. Generalizability of the study, which covered three regions of one malaria-endemic country, may have been reduced due to exclusion of HIV-positive children from the survey sample (based on SMC eligibility). Furthermore, we assumed a linear association between distance between households and health facilities, and health facility access, based on sensitivity analyses; however, it was not possible to verify whether this assumption held true for longer distances (> 20 km) due to the absence of relevant data.

## Conclusion and recommendations

The results provide insights into the existing situation regarding children’s access to healthcare facilities in Togo and its child, caregiver, household and area-level predictors. This study highlights the need for further efforts to facilitate children’s health facility access in Togo, including focusing on addressing socioeconomic barriers and improving proximity to health facilities by enhancing both transportation and health infrastructure. Further research, both qualitative and quantitative, is needed to understand the processes underlying barriers to healthcare access for febrile children, as has previously been elaborated for other groups such as pregnant women. Enhancing community education and engagement, including through leveraging communication and mobile health technologies, and promoting universal health coverage by removing financial barriers and promoting equity in service provision, may prove instrumental in addressing common challenges impeding healthcare access. More specifically, this study identified “fever considered not serious”, and “caregiver preferred alternative treatment” among the three most commonly reported by caregivers for lack of health access among febrile SMC-eligible children; our results highlight the potential for sensitization activities, both within communities and health facilities, to address these reasons directly by promoting preferences for effective malaria medicines among caregivers of young children, and educating caregivers on situations where medical attention by trained practitioners should be sought. Our findings can inform evidence-based policies and interventions for improved child health outcomes, particularly for those affected by malaria.

## Data Availability

The data that support the findings of this study, obtained from end-of-round SMC coverage surveys, are property of Malaria Consortium but may be made available to researchers for non-commercial purposes upon reasonable request to the corresponding author.

## Abbreviations

AQ: Amodiaquine
CI: Confidence interval
COVID-19: Coronavirus disease (2019)
HIV: Human immunodeficiency virus
AOR: Adjusted odds ratio
PF: Plasmodium falciparum
P: p-value
Ref: Reference
SMC: Seasonal malaria chemoprevention
SP: Sulfadoxine-Pyrimethamine
SSA: Sub-Saharan Africa
KM: Kilometer
WHO: World Health Organization
